# Pregnant in a Pandemic: Mental Wellbeing and Associated Healthy Behaviors Among Pregnant People in California During COVID-19

**DOI:** 10.1007/s10995-023-03657-w

**Published:** 2023-04-08

**Authors:** Jennifer E. Phipps, Mackenzie D. M. Whipps, Indira D’Souza, Janine M. LaSalle, Leigh Ann Simmons

**Affiliations:** 1 Department of Human Ecology, Perinatal Origins of Disparities Center, University of California, Davis, 1 Shields Ave, Davis, CA 95616 USA; 2 Department of Medical Microbiology and Immunology, Perinatal Origins of Disparities Center, University of California, Davis, 1 Shields Ave, Davis, CA 95616 USA

**Keywords:** Pregnancy, Mental wellbeing, COVID-19, Healthy behaviors

## Abstract

**Introduction:**

Pregnancy is a time of increased vulnerability to mental health disorders. Additionally, the COVID-19 pandemic has increased the incidence of depression and anxiety. Thus, we aimed to assess mental health and associated healthy behaviors of pregnant people in California during the pandemic in order to contextualize prenatal well-being during the first pandemic of the twenty-first century.

**Methods:**

We conducted an online cross-sectional study of 433 pregnant people from June 6 through July 29, 2020. We explored 3 hypotheses: (1) mental health would be worse during the pandemic than in general pregnant samples to date; (2) first-time pregnant people would have worse mental health; and (3) healthy behaviors would be positively related to mental health.

**Results:**

Many of our participants (22%) reported clinically significant depressive symptoms and 31% reported clinically significant anxiety symptoms. Multiparous pregnant people were more likely to express worries about their own health and wellbeing and the process of childbirth than were primiparous pregnant people. Additionally, as pregnancy advanced, sleep and nutrition worsened, while physical activity increased. Lastly, anxious-depressive symptomology was significantly predictive of participant sleep behaviors, nutrition, and physical activity during the past week.

**Discussion:**

Pregnant people had worse mental health during the pandemic, and this was associated with worse health-promoting behaviors. Given that the COVID-19 pandemic and associated risks are likely to persist due to low vaccination rates and the emergence of variants with high infection rates, care that promotes mental and physical well-being for the pregnant population should be a public health priority.

**Supplementary Information:**

The online version contains supplementary material available at 10.1007/s10995-023-03657-w.

## Significance

Consistent with other studies, we found that anxiety, depression, and stress levels in our pregnant cohort were higher than reported pre-pandemic and that anxious-depressive symptomology predicted worse sleep, poorer nutrition, and less physical activity. New in this study was that these relationships were the same for primiparous and multiparous pregnant people, did not change with gestational age, and that while sleep and nutrition worsened throughout pregnancy, physical activity actually increased, regardless of anxious-depressive symptomology. Also new to this study was that multiparous pregnant people had more worries about their and their baby’s wellbeing than first time mothers.

**Supplementary Information:**

The online version contains supplementary material available at 10.1007/s10995-023-03657-w.

## Introduction

Pregnant people are at increased risk of mental health disorders, such as depression and anxiety, compared to the general population (Albert, 2015; Biaggi et al., 2016). Perinatal mental health disorders also are the most common complications of pregnancy, contribute significantly to maternal morbidity and mortality, and account for high health care expenditures. A number of hypotheses regarding susceptibility to perinatal mental health disorders have been proposed, ranging from environmental exposures to hormonal shifts to situational risk factors. For example, pregnancy is both an emotional and physical stressor, and pregnant people with different resource capacities may be more or less likely to experience depression, anxiety, or other mood disorders (Fairbrother et al., 2015; Howard et al., 2014; O'Hara & Wisner, 2014). Regardless of etiology, pharmacologic treatment for perinatal mental health disorders lags behind treatment for mental health disorders in the general population, in part due to concerns over the potential teratogenic or other negative effects on fetal development. Moreover, the stigma of having a perinatal mental health disorder is a significant barrier to parents receiving care, in part due to fears of their infants being removed from the home.

Perinatal mental health has received increasing attention in the last two decades, especially as new pharmacotherapies have emerged. However, the COVID-19 pandemic has changed the landscape of mental health in general, and for pregnant people specifically. Several studies have already shown adverse effects of the COVID-19 pandemic on multiple outcomes for women (Almeida et al., 2020) and pregnant people, particularly increased stress, depression, and anxiety (Akgor et al., 2021; Berthelot et al., 2020; Lebel et al., 2020; Moyer et al., 2740; Preis et al., 2020; Sun et al., 2020). The recommended COVID-19 mitigation behaviors, such as social distancing and limiting interactions to one’s immediate family or “pod”, have meant that the social supports typically recommended for individuals with mental health disorders—as well as pregnant people—are limited to nonexistent, or come with the price of risking COVID-19 infection. Moreover, as counties and states have waxed and waned on “stay-at-home” orders due to changes in COVID-19 case rates, navigating health care decisions has become more stressful. Specifically, concerns over health care access and availability and weighing the pros and cons of seeking care versus potential exposure risk, have dominated pregnant people’s decision-making considerations and added a stressor that did not previously exist. In fact, studies have shown that pregnant people have reduced visits to their healthcare providers due to 1) worry of COVID-19 infection (Shayganfard et al., 2020) and 2) healthcare institutions not allowing in-person visits or “elective” hospital procedures (Gross et al., 2020; Whipps et al., 2021). Fewer visits to providers mean that pregnant people are less likely to be screened for mental health disorders. Moving forward, telehealth screening may help fill this gap, but effectiveness needs to be specifically assessed in the prenatal population.

The purpose of this investigation was to better understand how the COVID-19 pandemic has affected the mental health and associated health behaviors of pregnant people in California. Although California is currently “open and mask-free,” vaccine uptake among pregnant people lags behind the general population, and the rise of more infectious COVID variants, such as the Omicron variant, is resulting in public health guidance that is more restrictive (Dougherty et al., 2021; Farinholt et al., 2021). Both depression and anxiety are highly correlated with health behaviors such as nutrition (Owen & Corfe, 2017; Pols, 2018), physical activity (Paluska & Schwenk, 2000; Peluso & Guerra de Andrade 2005) and sleep (Buysse, 2013; Krystal, 2012; Vgontzas et al., 2012), and these behaviors have been the targets of psychosocial interventions to prevent and mitigate perinatal mental health disorders. Pregnant people in general are at higher risk for poor nutrition (Bailey et al., 2019), inadequate physical activity (Evenson & Wen, 2010) and disrupted sleep (Christian et al., 2019); and the relationship between poor mental health during pregnancy and a lack of engagement in positive health behaviors is well-established (Alhusen et al., 2016; Baskin et al., 2015; Bodnar & Wisner, 2005). At least one study has found a relationship between the COVID-19 pandemic and poor health behaviors (Arora & Grey, 2020). Thus, we anticipated that pregnant people experiencing worse mental health would have worse health behaviors compared to pregnant people with better mental health.

We hypothesized that: (1) the mental health of pregnant people in California would be substantially worse during the COVID-19 pandemic than in general pregnant cohorts to date; (2) people experiencing pregnancy for the first time (primiparity) would have worse mental health due to the unknowns of childbirth; and (3) healthy behaviors, including sleep, physical activity, and nutrition, would be positively related to the mental health of our pregnant cohort and vary based on gestational age. Lastly, we explored whether primiparity moderated these relationships. This knowledge has the potential to guide how prenatal care providers support patients during the ongoing COVID-19 pandemic, as well as provide data to support evidence-based policymaking related to perinatal mental health care and COVID-19 public health mitigation strategies.

## Methods

### Participants

We performed a cross-sectional survey of 433 pregnant people in California from June 6 through July 29, 2020. Participants were primarily recruited through social media, and both English and Spanish versions of the survey were available. Pregnant people between the ages of 18 and 45 were eligible if they were currently residing in California; all participants provided informed consent online and the protocol and consent method were approved by the UC Davis Institutional Review Board.

### Data Quality

We used several strategies to ensure high quality data were collected from this web-based survey. We removed data from participants who did not reach the end of the survey instrument, completed the survey in less than 10 min, or attempted to take the survey multiple times. In all, 155 out of 588 participants were not included as a result of these quality checks. Sporadic missingness was handled using list-wise deletion.

### Measures

*Mental Health*. We examined mental health in several domains: (a) anxious symptomology; (b) depressive symptomology; (c) perceived stress levels; and (d) specific concerns in the context of pregnancy and birth. Anxious symptomology was coded from the shortened Generalized Anxiety Disorder Scale (GAD-2) (Nath et al., 2018). Depressive symptomology was coded from the shortened Patient Health Questionnaire (PHQ-2) (Levis et al., 2020). Perceived stress was coded from the Perceived Stress Scale (PSS-10) (Cohen et al., 1983). All three mental health scales are well-established for use in clinical and research settings as well as among pregnant people. We also scored overall Anxious-Depressive Symptomology by summing the scores on the PHQ-2 and the GAD-2 for each participant, as was done similarly by Kroenke, et. al. (Kroenke et al., 2016). We used the shorter versions of the GAD and PHQ to keep the survey a reasonable length and because we were assessing risk for anxiety and depression rather than actually making a diagnosis.

To assess concerns regarding pregnancy and birth, we created 3 additional scales for analysis: (1) Worries about Pregnant Person; (2) Worries about Baby; and (3) Worries about Childbirth. All items in these three scales utilized response options on a Likert-type, behaviorally anchored scale for how often the participant was concerned about each specific issue. Responses ranged from 0 to 4: 0—*Never, 0 days*; 1—*Rarely, 1–2 days*; 2—*Sometimes, 3–4 days*, 3—*Often, 5–6 days*, and 4—*Always, 7 days*. Participants also had the option to select “*Does not apply*” and “*I prefer not to answer*”. The scales were created by summing the items within each category and were treated as continuous in analysis. The 3 items within the Worries about Pregnant Person scale include: “I worry about getting sick”, “I worry that I will be admitted to the hospital” and “I worry that I’m going to die”. The 5 items in the Worries about Baby scale include: “I worry about losing my pregnancy”, “I worry that my baby will be born early”, “I worry about my baby getting sick”, “I worry that my baby will have to stay in the hospital after he or she is born”, and “I worry that my baby will die”. The 4 items in the Worries about Childbirth include: “I worry that I will not have my birth support person with me in the delivery room”, “I worry that my provider or health care team will not be available during my delivery”, “I worry that my healthcare team will not have the equipment and resources they need to support my delivery”, and “I am thinking about not having my baby in a hospital”.

*Healthy Behaviors*. Healthy behaviors, including nutrition, physical activity, and sleep patterns, were operationalized using a similar method to the concern scales above. Individual items within each of these three scales can be found in the Appendix. Participants were again asked to report on the frequency of these behaviors (from Never to Always, 0–4) during the past 7 days. The Nutrition Scale has 4 items; the Sleep Scale has 7 items; and the Physical Activity Scale has 3 items.

*Primiparity and other Demographic Characteristics*. Primiparity, or being a first-time pregnant person, was treated as dichotomous in our analysis and was the moderator of interest. Some models described below also adjusted for other demographic features that may confound our analyses. These included: (a) participant age (in years), (b) urbanicity (on a 1–5 scale from *rural* to *major metropolitan*), (c) financial insecurity (assessed on a 1–5 scale from *No difficulty at all paying bills in the past 2 months* to *A great deal of difficulty paying bills in the past 2 months*), (d) ethnicity (Hispanic or Non-Hispanic, treated dichotomously in analysis), and (e) racial minoritization (treated categorically in analysis with 6 categories: white, Black/African American, Indigenous/First Nations, Asian/Asian American, Pacific Islander/Native Hawaiian, and multiracial).

### Data Analysis

To test our specific hypotheses regarding mental health, healthy behaviors, primiparity, and gestational age, we undertook several analyses. First, we described the mental health status of the sample across both primiparous and multiparous pregnant people, testing to see if mental health (both established scales and newly created ‘worries’ scales) varied by parity or gestational age using 2-tailed independent samples t-tests. Likewise, we investigated whether anxious-depressive symptomology predicted healthy behaviors across the three healthy behavior scales, controlling for confounding demographic covariates using a main effects multivariate ordinary least-squares (OLS) model. Finally, we assessed whether primiparity moderated these relationships by testing the interaction of anxious-depressive symptomology and primiparity on these same outcomes of interest, controlling for confounding covariates.

## Results

The demographics of our cohort are shown in Table 1. A substantial portion of the sample was under significant mental health strain: 22% of participants reported clinically significant depressive symptoms (PHQ-2 ≥ 3), and 31% reported clinically significant anxiety symptoms (GAD-2 ≥ 3). Further, 11% of the participants reported high levels of acute stress, with another 62% reporting moderate acute stress, and only 27% reporting low stress levels. However, across these three established scales, no statistical difference in depression, anxiety, or acute stress between primiparous and multiparous people or based on gestational week of pregnancy (see Table 2) was found.

**Table 1:** Demographic Characteristics, by Anxiety and Depression Clinical Cutoff Category

		Full Sample		PHQ-2 ≥ 3		GAD-2 ≥ 3	
		n = 433		n = 96		n = 135	
Characteristic		N	(%)	N	(%)	N	(%)
Maternal age							
18–24		53	(12.2)	22	(22.9)	25	(18.5)
25–34		255	(58.9)	54	(56.3)	84	(62.2)
35 +		125	(28.9)	20	(20.8)	26	(19.3)
Ethnicity							
Hispanic		149	(34.4)	36	(38.3)	45	(34.1)
Not Hispanic		280	(64.7)	58	(61.7)	87	(65.9)
Race							
White		233	(53.8)	43	(44.8)	71	(52.6)
Black/African American		20	(4.6)	6	(6.3)	6	(4.4)
Indigenous/First Nations		4	(.9)	1	(1.0)	2	(1.5)
Asian		35	(8.1)	10	(10.4)	8	(5.9)
Pacific Island/Native Hawaiian		1	(.2)	0	(.0)	0	(.0)
Other race		37	(8.6)	12	(12.5)	11	(8.2)
Multiracial		82	(18.9)	19	(19.8)	33	(24.4)
Urbanicity							
Rural		22	(5.1)	7	(7.5)	11	(8.4)
Semi-rural		51	(11.8)	21	(22.6)	20	(15.3)
Suburban		191	(44.1)	34	(36.6)	56	(42.8)
Urban		99	(22.9)	24	(25.8)	31	(23.7)
Major metropolitan		63	(14.6)	7	(7.5)	13	(9.9)
Financial Insecurity							
No difficulty paying bills		206	(47.6)	22	(22.9)	49	(36.6)
A little difficulty paying bills		80	(18.5)	20	(20.8)	22	(16.4)
Some difficulty paying bills		71	(16.4)	22	(22.9)	30	(22.4)
Quite a bit of difficulty paying bills		38	(8.7)	10	(10.4)	15	(11.2)
A great deal of difficulty paying bills		36	(8.3)	22	(22.9)	18	(13.4)
Parity							
Primipara		206	(47.8)	48	(50.0)	64	(47.4)
Multipara		227	(52.4)	48	(50.0)	71	(52.6)

Due to sporadic missingness, not all categories sum to 100%

**Table 2:** Mental health and worries by primiparity

	Primiparous	Multiparous	
	(n = 206)	(n = 225)	T-test
Established Mental Health Scales	N (%)	N (%)	Sig
PHQ-2			
Above Clinical Cutoff	48 (23.4)	48 (21.5)	
Below Clinical Cutoff	157 (76.6)	175 (78.5)	
GAD-2			
Above Clinical Cutoff	64 (31.1)	71 (31.3)	
Below Clinical Cutoff	142 (68.9)	156 (68.7)	
PSS (Stress Scale)			
High	27 (13.1)	22 (9.7)	
Moderate	118 (57.3)	149 (65.6)	
Low	61 (29.6)	56 (24.7)	
Newly Created Worries Scales for People	Mean (SD)	Mean (SD)	Sig
Worries about person (0–12)	3.82 (2.5)	4.36 (2.8)	*
Worries about fetal / infant wellbeing (0–20)	7.83 (4.4)	7.93 (5.0)	
Worries about childbirth (0–16)	4.43 (3.1)	5.09 (3.2)	*

Two-tailed independent samples t-tests determined whether primiparous participants differed significantly from multiparous participants

* p < 0.05, ** p < 0.01, *** p < 0.001

We similarly found that participants reported substantial worry regarding pregnancy and childbirth, specifically. Multiparous pregnant people were more likely to express worries about their own health and wellbeing and the process of childbirth than were primiparous pregnant people in our sample (Table 2). However, no differences in these worries based on gestational week of pregnancy existed. Conversely, healthy behaviors did change with increasing pregnancy weeks. Sleep and nutrition worsened with gestational age, while physical activity increased.

Using multivariate OLS models to control for participant age, urbanicity, financial insecurity, essential worker status, racial minoritization, and ethnic minoritization, we found that anxious-depressive symptomology was significantly predictive of participant sleep behaviors, nutrition, and physical activity during the past week (Table 3). However, the overall effect sizes were small across all three outcomes of interest. When primiparity was added as a moderator to these models, we saw no moderation of the effect size or significance of the main effects model: anxious-depressive symptomology was equivalently predictive of healthy behaviors for primiparous and multiparous participants (Table 4).

**Table 3:** Association between Anxious-Depressive Symptomology and Health Behaviors during COVID-19 (n = 433)

Outcome	β	(Std. Err.)	p-value	Sig	Cohen's D	
Sleep Scale	− 0.57	(.1)	< 0.001	***	− 0.11	
Nutrition	− 0.22	(.05)	< 0.001	***	− 0.08	
Physical Activity	− 0.29	(.06)	< 0.001	***	− 0.04	

Regression analyses control for participant age, urbanicity, financial insecurity, race, and ethnicity

**Table 4:** Association between Anxious-Depressive Symptomology and Health Behaviors, Moderated by Primiparity (n = 433)

Outcome	β	(Std. Err.)	p-value	Sig
Sleep Scale				
Anxious-Depressive Symptoms	− 0.73	(.14)	0.000	***
Primiparity	− 0.02	(.9)	0.979	
Primiparity*AD Symptoms	0.29	(.19)	0.127	
Nutrition				
Anxious-Depressive Symptoms	− 0.26	(.07)	0.000	***
Primiparity	0.05	(.46)	0.912	
Primiparity*AD Symptoms	0.07	(.1)	0.465	
Physical Activity				
Anxious-Depressive Symptoms	− 0.32	(.08)	0.000	***
Primiparity	− 0.35	(.52)	0.494	
Primiparity*AD Symptoms	0.06	(.11)	0.615	

Regression analyses control for maternal age, urbanicity, financial insecurity, race, and ethnicity

## Discussion

The purpose of this study was to examine mental wellbeing, including anxiety, depression, and pregnancy worries, and the health-promoting behaviors of sleep, diet, and physical activity in the context of the COVID-19 pandemic and pregnancy status. Consistent with other studies, we found that anxiety, depression, and stress were higher than reported pre-pandemic (Akgor et al., 2021; Almeida et al., 2020; Berthelot et al., 2020; Lebel et al., 2020; Moyer et al., 2740; Preis et al., 2020; Sun et al., 2020) and that anxious-depressive symptomology predicted worse sleep, poorer nutrition, and less physical activity (Buysse, 2013; Krystal, 2012; Owen & Corfe, 2017; Paluska & Schwenk, 2000; Peluso & Guerra de Andrade 2005; Pols, 2018; Vgontzas et al., 2012). New in this study was that these relationships were the same for primiparous and multiparous pregnant people, did not change with gestational age, and that while sleep and nutrition worsened throughout pregnancy, physical activity actually increased, regardless of anxious-depressive symptomology. Based on the known relationship between mental health and healthy behaviors, this does suggest that for pregnant people, increasing their physical activity, in comparison to targeting their nutrition or sleep, is a potential pathway to improving their anxious-depressive symptomology, particularly during a pandemic. However, these findings also suggest that sleep and nutrition may be important targets for improvement in future studies, given that they worsened as pregnancy advanced in our cohort and given data that show a strong relationship between these health-promoting behaviors and both maternal and fetal outcomes (Mate et al., 2021; Micheli et al., 2011; Palagini et al., 2014; Stang & Huffman, 2016; Warland et al., 2018).

In our subset of the pregnant population in California, we did find that overall, the cohort participated in healthy behaviors a moderate proportion of the time (see Appendix). No statistically significant differences between demographics or parity for the rates at which the cohort reported to participate in the healthy behaviors were found. However, in California, healthy behavior participation is likely different than in other parts of the country, so the theory that targeting improving health behaviors, particularly physical activity, to improve mental health in pregnancy should be validated in a larger, national cohort of pregnant people to verify that these results are not specific to only Californian pregnant people. Additionally, our participants were recruited via social media, and there is a known association between social media coverage of the pandemic and heightened stress and anxiety (He et al., 2020; Saha et al., 2020). Thus, as social media consumers, our participants may have been influenced by this negative association. However, most people in the United States are social media users, particularly women of childbearing age (Social Media Fact Sheet | Pew Research Center, 2021), and thus we hypothesize that all participants were equally affected within a small margin of error.

Contrary to our hypothesis, we found that multiparous pregnant people were more likely to worry about their health and childbirth than primiparous pregnant people. This may be due to the influence of previous birthing experiences; since these participants have experienced childbirth before, they may be more aware of the potential challenges that health care during a pandemic presents (e.g., limited visitation, care of a newborn with little or no family help, previous potentially traumatic births, etc.) than first time parents. Additionally, pregnant people with other children likely have higher levels of stress and anxiety overall, particularly during the timeframe when they responded to this survey due to many childcare centers and schools being closed, worries about keeping other children healthy, and the inability of parents to truly isolate from their children if they experienced a COVID-19 infection. Also, pregnant people in general have worse sleep than they did prior to pregnancy, irrespective of mental health and the COVID-19 pandemic (Sedov et al., 2018). Future studies should examine more pregnancy-specific sleep-factors, such as whether sleep is being impacted by fetal movement, discomfort due to the growing fetus, or frequent need to urinate, to better elucidate the relationship between poor mental health and poor sleep.

While the potential negative impacts of COVID-19 on perinatal mental health are clear, the pandemic also may provide a unique window into how we might leverage new knowledge about perinatal mental health disorders to inform health care policy, planning, and decision-making. For example, within the general US population, transparency about poor mental health has increased as a result of the pandemic (Almeida et al., 2020; Javed et al., 2020). More openness about mental health status and treatment has been shown to reduce the stigma associated with mental health disorders, and pregnant people may benefit from this changing social landscape (Beers & Joshi 2020; Clement et al., 2013; Collins et al., 2019; Griffiths et al., 2014). Additionally, many of the structural determinants of COVID-19 infection as well as morbidity and mortality, including poverty, housing and food insecurity, racism, and limited education are the same social determinants of increased risk for perinatal mental health disorders (Biaggi et al., 2016; Fernandez Turienzo et al., 2021; Linares et al., 2020). The pandemic has highlighted how desperately the US needs significant policy and programmatic changes to address structural barriers to good health and wellness for all people—and pregnant people will benefit from such changes. Lastly, significant work is still needed to increase rates and effectiveness of screening pregnant people for modifiable pregnancy risks. Currently, the American College of Obstetrics and Gynecology recommends one prenatal and one postnatal depression/anxiety screening, along with a succession of physical tests (e.g., sexually transmitted infections, chromosomal abnormalities, anemia) (ACOG Committee Opinion No, 2018; Learman, 2018; Levy et al., 2019; Screening for Fet al. & Chromosomal Abnormalities, 2020). However, a number of risk factors and structural determinants of health are associated both with poor perinatal mental health and poor pregnancy outcomes that could be screened and addressed (e.g., screening for mental disorders across trimesters, food insecurity and dietary intake, sleep disorders, and physical activity) (Andermann, 2018). The pandemic has brought to light the need to focus on the mental health of pregnant people and the relationship between mental health and structural determinants of health. This study has provided an example of how the pandemic has specifically negatively impacted the mental health of pregnant people. A significant gap exists between the need for mental health care access, availability, and affordability, and state and federal policies to improve these. Additionally, policies that address structural determinants are lagging, and economic supports that were made available through COVID emergency funds will end. Lessons learned from the pandemic in terms of how structural supports mitigated the impact of these determinants on mental health should be applied to current and future policymaking to improve the mental well-being of pregnant people and their infants.

Postpartum depression and anxiety are very common morbidities of pregnancy (Henshaw, 2003; Pearlstein et al., 2009; Ross & McLean, 2006; Shorey et al., 2018) and closely linked to mental health during pregnancy (Guintivano et al., 2018; Witt et al., 2011). A significant gap remains in the evidence regarding the etiology of perinatal mental disorders and the degree to which these disorders are biological, psychosocial, or biopsychosocial in origin. The most common biomarkers of maternal psychosocial stress in pregnancy are the hypothalamic–pituitary–adrenal (HPA) axis glucocorticoids that include corticotrophin-releasing hormone and the downstream release of cortisol in maternal blood and other tissues (Monk et al., 2019; Padula et al., 2020). However, interindividual differences in glucocorticoids and the daily rhythmicity of these HPA responses make cortisol levels a challenging biomarker in human studies (Almeida et al., 2009; Oster et al., 2017) and the mechanistic connection of cortisol levels to mental health states are tenuous (Monk et al., 2019; Staufenbiel et al., 2013). In contrast, epigenetic biomarkers of perinatal stress examine DNA methylation patterns at the interface of genetics and environmental stressors, so have the potential to explain interindividual differences in stress and multi-variate nature of perinatal stress from a variety of psychosocial influences over generations (Breton et al., 2021; Hong et al., 2021). For instance, a genome-wide study identified differences in DNA methylation at genes regulating immune responses in maternal samples associated with stress from a natural disaster (Cao-Lei et al., 2014). Furthermore, pregnancy associated DNA methylation biomarkers at immune markers of inflammation were predictive of anxiety and depression symptoms in response to discrimination (Sluiter et al., 2020). Understanding the biological impacts of the pandemic on mental health during pregnancy may help to elucidate the etiology, especially for people of color, who have been disproportionately affected by both the pandemic (Alcendor, 2020; Bibbins-Domingo, 2020; Cyrus et al., 2020; Macias Gil et al., 2020; Mackey et al., 2020; Millett et al., 2020) and poor pregnancy outcomes (Gur et al., 2020; Minkoff, 2020).

In addition to affecting long-term mental health outcomes of pregnant people, perinatal stress has been associated with neurodevelopmental and neuropsychiatric outcomes of the offspring (Dunkel Schetter & Tanner, 2012; Manzari et al., 2019). The additional perinatal stressors associated with the COVID-19 pandemic are therefore important considerations for the interpretation of child outcomes from prospective and retrospective birth cohort studies that include births in the years 2020–2021. For instance, the National Institutes of Health’s Environmental Influences on Child Health Outcomes (ECHO) program, which was already planning to examine the additive effects of perinatal stress and environmental chemical exposures on child health outcomes (Padula et al., 2020), has added additional questionnaires similar to the ones used here to measure COVID-19 pandemic-related perinatal stress. Biomarkers of stress including glucocorticoid and DNA methylation measurements are also planned for pregnancy cohorts conducted during the COVID-19 pandemic and may reveal further insights into the mechanisms into interindividual differences of offspring health outcomes in response to pandemic-related perinatal stress. Together, these studies are expected to reveal new insights into individual differences in response to risk and resiliency of the impact of pandemic related stress during pregnancy.

## Conclusion

This study corroborates previous research that has shown worsening mental health due to the pandemic among a pregnant cohort in California. Additionally, this work shows the link between mental health and healthy behaviors, suggesting the importance of improving pregnant people’s mental and physical wellbeing through improved screening for mental health status and prenatal provider education regarding how to improve sleep, nutrition, and physical activity. Leveraging focused efforts to improve prenatal mental and physical health during the pandemic may extend into the post-pandemic landscape, helping to improve pregnancy health and outcomes, postpartum mood disorders, and addressing the gap that exists between need for mental health care availability, access, and affordability.

## Supplementary Information

Below is the link to the electronic supplementary material.

Supplementary file1 (DOCX 17 KB)

## Data Availability

Data available upon reasonable request.
