# Supplementary material for: Pregnant in a Pandemic: Mental Wellbeing and Associated Healthy Behaviors Among Pregnant People in California During COVID-19
Source: Matern Child Health J. 2023 Apr 8;27(7):1254–63. doi: 10.1007/s10995-023-03657-w (PMC10083068; doi:10.1007/s10995-023-03657-w)
Supplement: Supplementary file 1 — Supplementary file1 (DOCX 17 KB) [file 10995_2023_3657_MOESM1_ESM.docx]

| Appendix 1: Items in Healthy Behaviors Scales | |  |  |  |  |  |  |  |  |  |  |
| --- | --- | --- | --- | --- | --- | --- | --- | --- | --- | --- | --- |
|  |  | Never | | Rarely | | Sometimes | | Often | | Always | |
|  |  | N | % | N | % | N | % | N | % | N | % |
| Physical Activity | |  |  |  |  |  |  |  |  |  |  |
|  | I make sure to get outside for at least 30 minutes | 15 | 3% | 73 | 17% | 132 | 30% | 114 | 26% | 97 | 22% |
|  | I get at least 30 minutes of moderate activity | 45 | 10% | 83 | 19% | 140 | 32% | 104 | 24% | 59 | 14% |
|  | I get at least 30 minutes of vigorous activity | 134 | 31% | 128 | 30% | 99 | 23% | 36 | 8% | 33 | 8% |
| Nutrition |  |  |  |  |  |  |  |  |  |  |  |
|  | I eat sweets * | 17 | 4% | 111 | 26% | 159 | 37% | 109 | 25% | 34 | 8% |
|  | I eat salty snacks * | 21 | 5% | 116 | 27% | 193 | 45% | 77 | 18% | 23 | 5% |
|  | I pay attention to healthy nutrition when I make food choices | 11 | 3% | 32 | 7% | 134 | 31% | 166 | 38% | 88 | 20% |
|  | I drink sugary drinks * | 125 | 29% | 146 | 34% | 100 | 23% | 40 | 9% | 16 | 4% |
| Sleep |  |  |  |  |  |  |  |  |  |  |  |
|  | My sleep has been interrupted * | 44 | 10% | 83 | 19% | 110 | 25% | 117 | 27% | 75 | 17% |
|  | I am sleeping less than usual * | 112 | 26% | 96 | 22% | 96 | 22% | 70 | 16% | 53 | 12% |
|  | I am sleeping more than usual * | 107 | 25% | 135 | 31% | 94 | 22% | 55 | 13% | 32 | 7% |
|  | I wake up frequently in the middle of the night * | 31 | 7% | 87 | 20% | 88 | 20% | 112 | 26% | 112 | 26% |
|  | It takes me 15 minutes or more to fall asleep at night * | 44 | 10% | 81 | 19% | 104 | 24% | 77 | 18% | 122 | 28% |
|  | I sleep at least 7 hours every night | 17 | 4% | 72 | 17% | 93 | 21% | 133 | 31% | 117 | 27% |
|  | I feel rested when I wake up | 40 | 9% | 105 | 24% | 154 | 36% | 105 | 24% | 28 | 6% |
| * Reverse coded when summed in scales | |  |  |  |  |  |  |  |  |  |  |
